# Sexual health risk reduction interventions for people with severe mental illness: a systematic review

**DOI:** 10.1186/s12889-015-1448-4

**Published:** 2015-02-12

**Authors:** Abdullah Pandor, Eva Kaltenthaler, Agnes Higgins, Karen Lorimer, Shubulade Smith, Kevan Wylie, Ruth Wong

**Affiliations:** Health Economics and Decision Science, ScHARR, University of Sheffield, Sheffield, UK; School of Nursing and Midwifery, Trinity College Dublin, Dublin, Ireland; School of Health and Life Sciences, Glasgow Caledonian University, Glasgow, UK; Institute of Psychiatry at Kings College London, London, UK; Porterbrook Clinic, Sheffield, UK

**Keywords:** Systematic review, Schizophrenia, Bipolar disorder, Sexual behavior, Sexually transmitted diseases, Sexuality

## Abstract

**Background:**

Despite variability in sexual activity among people with severe mental illness, high-risk sexual behavior (e.g. unprotected intercourse, multiple partners, sex trade and illicit drug use) is common. Sexual health risk reduction interventions (such as educational and behavioral interventions, motivational exercises, counselling and service delivery), developed and implemented for people with severe mental illness, may improve participants’ knowledge, attitudes, beliefs behaviors or practices (including assertiveness skills) and could lead to a reduction in risky sexual behavior. This systematic review evaluates the effectiveness of sexual health risk reduction interventions for people with severe mental illness.

**Methods:**

Thirteen electronic databases (including MEDLINE, EMBASE and PsycINFO) were searched to August 2014, and supplemented by hand-searching relevant articles and contacting experts. All controlled trials (randomized or non-randomized) comparing the effectiveness of sexual health risk reduction interventions with usual care for individuals living in the community with severe mental illness were included. Outcomes included a range of biological, behavioral and proxy endpoints. Narrative synthesis was used to combine the evidence.

**Results:**

Thirteen controlled trials (all from the USA) were included. Although there was no clear and consistent evidence that interventions reduce the total number of sex partners or improved behavioral intentions in sexual risk behavior, positive effects were generally observed in condom use, condom protected intercourse and on measures of HIV knowledge, attitudes to condom use and sexual behaviors and practices. However, the robustness of these findings is low due to the large between study variability, small sample sizes and low-to-moderate quality of included studies.

**Conclusions:**

There is insufficient evidence at present to fully support or reject the identified sexual health risk reduction interventions for people with severe mental illness. Given the serious consequences of high-risk sexual behaviors, there is an urgent need for well-designed UK based trials, as well as training and support for staff implementing sexual health risk reduction interventions.

**Trial registration:**

PROSPERO CRD42013003674.

**Electronic supplementary material:**

The online version of this article (doi:10.1186/s12889-015-1448-4) contains supplementary material, which is available to authorized users.

## Background

Severe mental illness (SMI), such as schizophrenia and bipolar disorder [1], persist over time and can result in extensive disability leading to impairments in social and occupational functioning [2]. Schizophrenia is estimated to affect approximately 180,471 [3] to 220,000 [4] people in the UK and bipolar disorder approximately 136,440 [3] to 297,000 [5]. While some individuals have long periods during which they are well and are able to manage their illness, many individuals with SMI have difficulties in establishing stable social and sexual relationships [6]. Despite variability in sexual activity among people with SMI (for example, people with schizophrenia-spectrum disorder are less likely than those with other major psychiatric disorders to be sexually active) [2], high-risk sexual behavior (e.g. unprotected intercourse, multiple partners, sex trade and illicit drug use) is common [2,7,8] and rates of blood borne viruses, such as HIV and Hepatitis C, have been found to be higher among people with SMI [9] (including those who are homeless and/or have a substance misuse problem) [10,11] than the general population. Risk behaviors for HIV among people with SMI can be influenced by substance use, childhood abuse, social relationships, and cognitive–behavioral factors [2,12].

The US Centers for Disease Control and Prevention (CDC) list best- and good-level interventions for HIV prevention in the “Compendium of Evidence-Based HIV Behavioral Interventions”, and continue to update this in light of new effectiveness evidence [13]. Behaviorally focused interventions have long been at the forefront of HIV prevention [14] and are characterized by their complexity and inclusion of multiple components. Behavioral strategies, such as those which attempt to delay onset of first intercourse, decrease the number of sexual partners or provide counselling and testing for HIV, can be focused at the level of individuals, couples, families and peer groups [15]. Interventions with, for example, an educational, behavioral, and/or counselling element could be developed and implemented for people with SMI, which may lead to improved participants’ knowledge, attitudes, beliefs or behavioral practices (including assertiveness skills), which could lead to a reduction in risky sexual behavior [8]. However, this would require an understanding of the range of risk behaviors among those with SMI and an assessment of the effectiveness of interventions.

The aim of this research was to systematically review the evidence on the effectiveness of sexual health risk reduction interventions for people with SMI compared with usual care.

## Methods

A systematic review was undertaken in accordance with the general principles recommended in the Preferred Reporting Items for Systematic Reviews and Meta-Analyses (PRISMA) statement [16].

### Data sources and searches

Potentially relevant studies were identified through searches of thirteen electronic databases and research registers including MEDLINE (1948 to August 2014), EMBASE (1980 to August 2014), CINAHL (1982 to August 2014) and PsycINFO (1806 to August 2014). The search strategy used free text and thesaurus terms and combined synonyms relating to the condition (e.g. schizophrenia, psychotic disorders, bipolar disorder and severe mental illness) with terms for sexual health interventions (e.g. sexual behavior, sexually transmitted diseases and sexual health). A methodological filter aimed at restricting search results to controlled trials was used in the searches of MEDLINE, EMBASE, CINAHL and PsycINFO. Date and language restrictions were not used on any database (see online Additional file 1). Searches were supplemented by hand-searching relevant articles (including citation searching), systematic keyword searches of the World Wide Web and mental health organization websites and contacting experts in the field (including authors of three ongoing studies).

### Study selection

Initially, all titles were examined by one reviewer. Citations that clearly did not meet the inclusion criteria were excluded i.e. non-human, unrelated to sexual health risk reduction interventions for people with SMI. Then, all abstracts and full text articles were examined independently by two reviewers. Any disagreements in the selection process were resolved through discussion. The systematic review included all controlled trials (randomized or non-randomized). Before and after studies without a concurrent control group were excluded because the absence of a control group to record concurrent changes over time means that changes due to the intervention or due to temporal trends, concurrent changes or a Hawthorne effect (a process in which subjects in a study change their behavior or performance in response to being observed) would be conflated. Such studies therefore represent very weak evidence of effectiveness [17,18]. Studies from developing countries were also excluded as it is difficult to generalize (e.g. transferability and acceptability) the characteristics of the effective interventions to developed countries. Eligible studies were those that included adult patients (aged over 18 years) with SMI living in the community. The term SMI usually refers to a severe and enduring mental illness associated with functional impairment that typically involves psychosis (losing touch with reality characterized by experiencing delusions and/or hallucinations) and commonly includes a diagnosis of schizophrenia, other psychoses and bipolar disorder [1]. In studies where there was a mixture of diagnostic groups, only those studies where the majority of participants (that is, more than 50%) had psychotic diagnosis were included. We did not include studies where the sole diagnosis was major depression. Any health promotion intervention or combination of interventions (e.g. educational, behavioral, psychological, counselling etc. delivered at the individual, group and community level) intended to change the sexual knowledge, attitudes, beliefs, behaviors or practices of individuals and populations to improve their sexual health outcomes (by avoiding high risk sexual behavior through enhancing skills in, for example, decision making for safe sex and assertiveness with the aim of reducing adverse biological outcomes e.g. sexually transmitted diseases and HIV) were included. Interventions that focused on sexual dysfunction and sexual violence or sexual dysfunction attributable to the use of prescribed medications were excluded. The comparator was considered as standard usual care in the community and the main sexual health related outcomes included a range of biological (sexually transmitted infections including HIV, unintended pregnancy), behavioral (numbers of partners, use of contraception/condoms, uptake of screening or treatment services) and proxy (knowledge, attitudes and beliefs, barriers and facilitators, intentions, skills) endpoints.

### Data abstraction and assessment of methodological quality

Data relating to study design, methodological quality, and outcomes, were extracted by one reviewer into a standardized data extraction form and independently checked for accuracy by a second. Where multiple publications of the same study were identified, data were extracted and reported as a single study. The study quality characteristics were assessed according to (adapted) criteria based on those proposed by the Effective Public Health Practice Project – EPHPP [19]. This is a generic tool used to evaluate a variety of intervention study designs such as controlled trials and observational studies. This tool has been judged suitable to be used in systematic reviews of effectiveness [20] and has been reported to have content and construct validity [19,21]. Consideration of study quality included the following six criteria: [1] selection bias - the extent to which study participants were representative of the target population [2] study design; [3] control of confounders; [4] blinding - whether outcome assessors, intervention providers and participants were aware of the research question [5] data collection methods and [6] withdrawals and dropouts. The six domain-based criteria were each rated as strong, moderate or weak depending on the characteristics of each criterion reported in the included study (Table 1). An overall assessment of study quality was based on the following ratings: studies with at least four criteria rated as ‘strong’ and with no criteria rated as ‘weak’, were given an overall rating of ‘strong’. Those studies receiving less than four ‘strong’ ratings and only one ‘weak’ rating were given an overall rating of ‘moderate’. A rating of ‘weak’ was given if two or more criteria were rated as ‘weak’. Additional study quality items included an assessment of intervention integrity, statistical analysis and generalizability to the UK. Any discrepancies in the data abstraction and quality assessment process were resolved through discussion to achieve agreement.

**Table 1:** **Quality assessment components and ratings for the EPHPP Instrument (Adapted with permission of the author) **[19]

**Components**	**Strong**	**Moderate**	**Weak**
Selection Bias	Very likely to be representative of the target population and participation rate >80%	Somewhat likely to pre representative of the target population and participation rate between 60 to 79%	All other responses or not stated
Study design	Randomized controlled trial or controlled clinical trial	Cohort analytic, case control, cohort or an interrupted time series	All other designs or design not stated
Confounders	Controlled for at least 80% of confounders	Controlled for at least 60 to 79% of confounders	Confounders not controlled for or not stated
Blinding	Blinding of outcome assessors and study participants to intervention status and/or research question	Blinding of either outcome assessors or study participants	Outcome assessors and study participants are aware of intervention status and/or research question
Data collection methods	Tools shown to be valid and reliable	Tools shown to be valid but reliability not described	No evidenced of validity or reliability
Withdrawals and dropouts	Participants completing study (or follow-up rate) >80%	Participants completing study (or follow-up rate) between 60 to 79%	Participants completing study (or follow-up rate) <60% or withdrawals and dropouts not described

### Data synthesis and analysis

A meta-analysis was not conducted on the data, as the studies were considered to be too heterogeneous with regards to the study designs, interventions and types of outcome data available. Therefore, as suggested by the guidance produced by the Cochrane Collaboration [22] and the Centre for Reviews and Dissemination (CRD) for undertaking systematic reviews [23,24], a narrative synthesis of included studies (grouped by outcome) was undertaken.

## Results

### Trial flow

**Figure 1:**
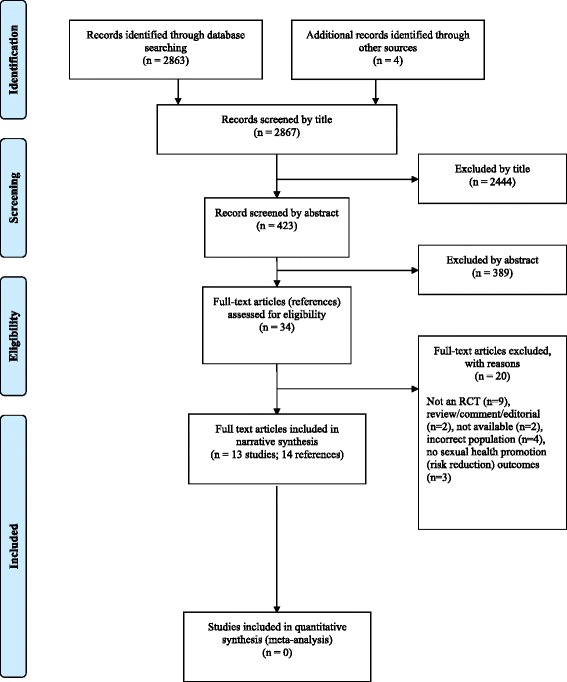
**Study flow chart (adapted).**

Figure 1 summarizes the process of identifying and selecting relevant literature. Of the 2867 citations identified, 13 studies (representing 14 references) [25-38] met the inclusion criteria.

### Study and patient characteristics

Full study details are presented in Table 2. All studies were published between 1996 and 2012 and were conducted in the USA (the majority of which were funded, at least in part, by the National Institute of Mental Health) [26,28-30,32,35-37]. Length of follow-up ranged from two weeks [31] to 18 months [36]. The content of the risk reduction interventions for improving sexual health varied between studies; however, most included HIV intervention programmes [28-32,35] (that focused on providing education to enhance HIV knowledge and skills training and or cognitive behavioral therapy) to prevent or reduce the risk of HIV and skills development to negotiate and practice safe sex including developing condom use skills [25,26,33,34,36,37]. Although no explicit details were provided on the delivery method in each study, the most common deliverers were trained facilitators [28-30,32,34,37,38], mental health counsellors [26] or mental health professionals [33,35,36]. The duration of the intervention sessions ranged from four [30,31] to 15 sessions [36]. Standard usual care included educational sessions on HIV [25,27,32,33,37], money management [26,29,36], HIV and substance misuse education [28], waiting list or no treatment [30,31,38], or health promotion covering a variety of topics [34,35].

**Table 2:** **Summary of study characteristics of controlled trials**

**Study, year**	**Setting and location**	**Population** ^**a**^	**Intervention**	**Control**	**Outcomes measured**	**Follow up**
Berkman et al. 2006, [25,27]^b^	Homeless shelter, New York, USA	Sample size: 92	Social skills training approach with cognitive-behavioral theory (6 sessions). Details unclear on who delivered the intervention.	Standard HIV (1 session) education (n = 42; of which 23 sexually active)	Unprotected anal, vaginal, oral sex with casual partners (women or men) as measured by VEE score^c^	6 months
		Mean age: 38 years				
		Male: 100%				
		Diagnosis: Schizophrenia or schizoaffective disorder, 72%; bipolar disorder, 3%; major depressive disorder, 10%	Intervention included videos, role-playing activities for development of skills, condom use skills, negotiating safer sex, behavior change, education on risks and problem solving skills (n = 50; of which 33 sexually active)			
		Ethnicity: African-American, 65%				
Berkman et al. 2007,[26]	Outpatient psychiatric clinics, New York, USA	Sample size: 149	Social skills training approach with cognitive-behavioral theory (10 sessions with boosters at 3, 6 and 9 months) delivered by substance abuse and/or mental health counsellors.	Money-management with matched treatment for dosage and format of the intervention group (n = 76)	Unprotected anal, vaginal, oral sex with casual partners (women or men) as measured by VEE score^c^	12 months
		Mean age: NR				
		Male: 100%				
		Diagnosis: Schizophrenia, 49%; schizoaffective disorder, 22.8%; bipolar disorder, 9.4%; major depressive disorder, 5.4%	Intervention included role-playing activities for development of skills, condom use skills, negotiating and practising safer sex (e.g. ethics, goals, commitment), behavior change, education on risks and problem solving skills (n = 73)			
		Ethnicity: African-American, 53.7%				
Carey et al. 2004,[28]^b^	Outpatient psychiatric clinics, New York, USA	Sample size: 408	HIV risk reduction programme (10 sessions) including enhancing knowledge about HIV transmission, and prevention, motivation for behavior change and strengthening behavioral skills and self-management training (n = 142)	Standard care which included HIV and substance use education, if needed (n = 126)	Frequency of unprotected vaginal sex, total number of sex partners, total number of casual partners, number of safer sex communications before intercourse and self-report of STIs	6 months
		Mean age: 36.5				
		Male: 46%				
		Diagnosis: Schizophrenia, 18%; schizoaffective disorder, 15%; bipolar disorder, 19%; major depressive disorder, 49%				
		Ethnicity: African-American, 21%	Substance use reduction programme (10 sessions) including enhancing knowledge, motivation and skills to reduce caffeine consumption, smoking, and alcohol use (n = 140)			
			All interventions delivered by trained clinical facilitators (with weekly supervision from a licensed clinical psychologist).			
Collins et al. 2011, [29]	Urban community setting, New York, USA	Sample size: 79	HIV prevention programme with social cognitive theory (10 sessions) delivered by trained facilitators (no further details provided).	Money-management (10 session workshop on managing finances and last through the month) (n = 40)	Unprotected anal, vaginal, oral sex with sexual partners (casual, steady, exchange) as measured by VEE score^c^	6 months
		Mean age: 42.3				
		Male: 0%				
		Diagnosis: Schizophrenia, 50%; schizoaffective disorder/ psychosis not specified, 14%; mood disorder with psychosis, 13%; mood disorder without psychosis, 23%	Intervention focus was on self-efficacy and skills training and included HIV/STI awareness, risk prevention, self-assertiveness, negotiating and practising safer sex, condom use skills; problem solving skills and commitment to self-protection (n = 39)			
		Ethnicity: Black, 61%				
Kalichman et al. 1995, [30]^b^	Outpatient psychiatric community care, Wisconsin, USA	Sample size: 52	HIV prevention programme based on behavioral skills training (4 sessions) delivered by trained facilitators experienced in HIV risk reduction interventions.	Waiting list group (who later received the intervention) (n = 29)	Knowledge, condom use, behavior change interventions	2 months
		Mean age: 39.2				
		Male: 52%				
		Diagnosis: Schizophrenia, 62%; schizoaffective disorder, 23%; major affective disorder including bipolar, 13%	Intervention included education on risk reduction, sexual assertiveness, negotiation skills (risk-related behavioral self-management), condom use and problem-solving skills (n = 23)			
		Ethnicity: African-American, 19%				
Katz et al. 1996,[31]^b^	Outpatient psychiatric centre, California, USA	Sample size: 27	AIDS education and risk reduction training programme (4 sessions). Details unclear on who delivered the intervention.	No treatment (n = 12)	Knowledge, behavior change interventions	2 weeks
		Mean age: NR				
		Male: NR but male female ratio 2:1				
		Diagnosis: NR but majority of patients diagnosed with schizophrenia and bipolar disorder	Intervention included education about HIV and AIDS, refusal skills training and problem solving skills (n = 15)			
		Ethnicity: NR				
Kelly et al. 1997,[32]^b^	Outpatient psychiatric care, Wisconsin, USA	Sample size: 104	Cognitive-behavioral therapy (7 sessions) that focused on behavior changes to reduce the risk of contracting HIV. Interventions included education on risk reduction, sexual assertiveness, negotiation skills (risk-related behavioral self-management), condom use and problem-solving skills (n = 34)	A single 60 minute AIDS education session (n = 28)	AIDS risk behavior (knowledge and safer-sex practices), and condom use: barriers to behavior change and perceived risk reduction, self-efficacy for use	3 months
		Mean age: 33.7				
		Male: 47%				
		Diagnosis: Schizophrenia, 19%; mood disorder, 58%; anxiety disorder, 11%; substance use or personality disorder, 11%				
		Ethnicity: African-American, 39%	Cognitive-behavioral therapy (7 sessions) combined with advocacy training (to act as a risk reduction advocate to their friends and acquaintances) (n = 42)			
			All interventions delivered by facilitators (no further details provided)			
Linn et al. 2003,[33]^b^	Homeless shelter, Nashville, USA	Sample size: 257	Social skills training approach with cognitive-behavioral theory (6 sessions) delivered by HIV educators, a mental health professional and a ‘paraprofessional’.	HIV and STI information (6 sessions) and basic instruction on condom use (n = 127)	Unprotected anal, vaginal, oral sex with casual, occasional and regular partners (women or men) as measured by VEE score^c^	6 months
		Mean age: NR				
		Male: 100%				
		Diagnosis: Schizophrenia/schizoaffective disorder, 61%; major depression/ bipolar disorder, 26%; other, 14%	Intervention included Sex, Games and Videotapes with storytelling, competitive games and acting scenes with true to life scenarios (n = 130)			
		Ethnicity: African-American, 54%				
Malow et al. 2012, [34]^b^	Outpatient psychiatric clinics, Florida, USA	Sample size: 290	Enhanced cognitive behavioral skill building programme (6 sessions) delivered by trained facilitators (no further details provided).	Health promotion including provision of information on HIV, heart attacks, good food habits, exercise, smoking and stress (n = 126)	HIV knowledge, perceived susceptibility, AIDS related anxiety, personal condom attitudes, peer and partner sexual attitudes, condom use skills, sexual self-efficacy, total number of unprotected vaginal sex acts, proportion of unprotected vaginal sex acts, total number of sex partners.	6 months
		Mean age: 39.6				
		Male: 45%				
		Diagnosis: schizophrenia, 15.7%; schizoaffective disorder, 8.4%; bipolar disorder, 9.6%; major depressive disorder, 21.2%	Intervention included HIV education, condom use, safe sex*,* high risk situations, and communication skills (n = 164)			
		Ethnicity: African-American, 55%				
NIMH 2006,[37]^b^	Outpatient mental health clinics, New York and Los Angeles, USA	Sample size: 99	Living in good health together programme (7 sessions) delivered by trained facilitators (no further details provided).	A single AIDS education session including video, discussion, and referral information (n = 47)	Number of partners; number of risky sexual acts, proportion of condom use; consistent condom use	12 months
		Mean age: NR				
		Male: 100%				
		Diagnosis: NR but patients with schizophrenia and bipolar disorder were eligible	Small group interventions covered knowledge of HIV, personal triggers for risk behavior, problem solving skills, condom use, assertiveness, negotiation strategies and relapse prevention (n = 52)			
		Ethnicity: African-American, 72.4%				
Otto-Salaj et al. 2001, [35]^b^	Outpatient mental health clinics, Wisconsin, USA	Sample size: 189	HIV prevention programme (7 sessions with boosters at 1 and 2 months later) delivered by trained mental health facilitators.	Health promotion including educational discussion and skills building exercises (focused on personal relationships, stress, nutritional health, cancer, heart disease and general sexual health) (n = NR)	HIV risk knowledge, attitudes towards condom use; risk reduction behavioral intentions; frequency of protected and unprotected intercourse; intercourse occasions protected by condoms; number of partners;	12 months
		Mean age: 38.4				
		Male: 46%				
		Diagnosis: Schizophrenia, 35%; affective disorder, 34%; schizoaffective disorder, 18%; other, 13%	Intervention included HIV risk reduction, condom use, problem solving strategies, discussion and role-play, negotiation and assertiveness skills and behavior change (n = NR)			
		Ethnicity: African-American, 51%				
Susser et al. 1998, [36]	Homeless men, New York, USA	Sample size: 59 (sexually active)	Social skills training approach with cognitive-behavioral theory (15 sessions) delivered by a mental health professional and a ‘paraprofessional’.	Health promotion (2 sessions) including provision of information on HIV, STI and condom use (n = 26)	Unprotected anal, vaginal, oral sex with casual and occasional partners (women or men) as measured by VEE score^c^	18 months
		Mean age: NR				
		Male:100%				
		Diagnosis: Schizophrenia/schizoaffective disorder, 61%; major depression/ bipolar disorder, 27%; other, 12%	Intervention included Sex, Games and Videotapes with storytelling, competitive games and acting scenes with true to life scenarios (n = 33)			
		Ethnicity: African-American, 58%				
Weinhardt et al. 1998, [38]^b^	Outpatient psychiatric care, New York, USA	Sample size: 20	Sexual assertiveness programme (10 sessions) delivered by a facilitator (no further details provided)	No treatment (n = 11)	Sexual assertiveness, knowledge, motivation, HIV risk behavior	4 months
		Mean age: 36				
		Male: 0%				
		Diagnosis: Schizophrenia spectrum disorders, 50%; bipolar disorder, 30%; major depressive disorder, 20%	Intervention included HIV related information and risk-behavior reduction, skill acquisition and fluency building and generalization of skills to actual interactions (n = 9)			
		Ethnicity: NR				

NR, not reported; STI, sexually transmitted infections; VEE, vaginal episode equivalent.

^a^Characteristics at baseline.

^b^Although all studies were described as RCTs by the study authors, this study did not report the method of randomization. According to the EPHPP quality assessment tool, this study would be categorized as a controlled clinical trial.

^*c*^The VEE score is a sexual behavior risk index. It is calculated using the following formula: (number of unprotected vaginal episodes) + (2 X number of unprotected anal episodes) + (0.1 X number of unprotected oral episodes). The VEE can be refined when data are extensive. For further details see Susser et al. [39].

Study populations were recruited from different settings and included homeless shelters [25,27,33,36], outpatient psychiatric clinics [26,28,30,32,35,37,38], residential facilities in a community setting [29], a drop-in socialization center [31] and a treatment programme for substance misusers [34]. Most studies included participants with a range of psychiatric diagnoses, which included schizophrenia, schizoaffective disorder, bipolar affective disorder and major depressive disorders. In one study [37], the psychiatric status and functioning of patients was not reported. For this study, it was assumed that people with mental health problems at high risk of HIV included some people with SMI. Sample sizes ranged from 20 [38] to 408 [28] patients, with mean participant age ranging from 33.7 [32] to 42.3 years [29] (data not reported in four studies) [31,33,36,37]. Five studies included males only [25-27,33,36,37], two women only [29,38] and the remaining studies included both men and women (range from 45% [34] to 52% male) [30]. There was wide variation in the ethnicity of participants between the studies, with seven studies reporting the majority of participants to be of African-American origin [25-27,33-37]. Although four studies [28,32,35,38] provided no information on comorbidities, coexisting problems such as alcohol and drug dependence were common.

### Quality assessment

**Figure 2:**
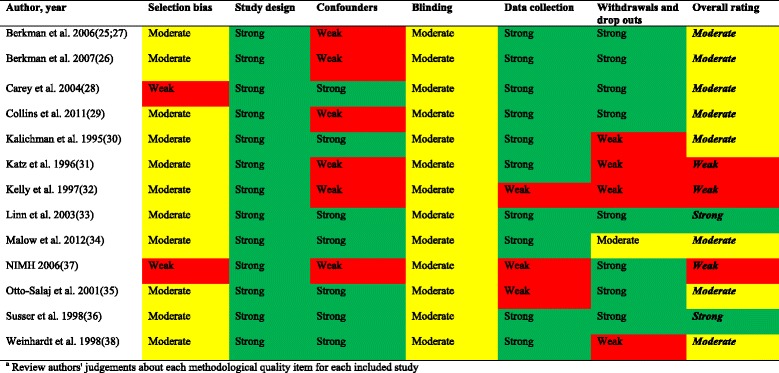
**Methodological quality summary** ^**a**^ **using the EPHPP Instrument** **[** 19 **]** **.**

A summary of the methodological quality of the included studies is presented in Figure 2. Generally, only two studies [33,36] were considered as having very few methodological limitations. All studies, except one [37], selected participants that were ‘somewhat likely’ to be representative of the target population; however, most studies (54%) did not report the number of individuals who were eligible to participate [25,26,30,34,35,37] or reported very low numbers of eligible individuals who agreed to participate in the study [28]. Although all the studies were described as RCTs, only three studies reported the method of randomization [26,29,36]. In seven studies confounders were well controlled; [28,30,33-36,38] however, in the remaining studies [25,26,29,31,32,37] no details on baseline compatibility were provided or if a variable was associated with the intervention or exposure and causally related to the outcome of interest. None of the studies were graded as ‘strong’ for blinding. Only five studies [26,28,33,34,36] blinded the outcome assessors and protected against detection bias. All studies failed to provide details on whether study participants were aware of the research question (reporting bias). Reliable and valid outcome measures were used in most (77%) of the studies [25,26,28-31,33,34,36,38]. While three studies [30,32,38] failed to provide details of withdrawals and dropouts, the follow-up rate was 80% or greater in eight studies [25,26,28,29,33,35-37]. Intervention integrity (assurance that the intervention was delivered according to plan) is an important part of program delivery. Only five studies [29,30,33,35,36] reported that more than 80% of participants received the allocated intervention and eight studies measured the consistency of the intervention [28-30,32,33,35-37], which was considered satisfactory. Contamination or co-intervention was unlikely in three studies [25,30,33], likely in one study [29] and not reported in nine studies [26,28,31,32,34-38]. No studies reported a sample-size calculation. Many of the studies had small sample sizes so it is likely they had inadequate statistical power to detect between group differences, even if they were present. The statistical analysis in most studies was appropriate and used intention-to-treat analysis. All the included studies were conducted in the USA, thus, making generalizability of the findings to the UK setting uncertain.

### Study results

#### Biological outcomes

No studies evaluated the incidence of unintended pregnancies. Only one study [28] indicated that participation in a small-group, 10 session HIV risk-reduction and substance misuse reduction intervention significantly reduced the likelihood of new sexually transmitted infections (self-reported) over six months compared with usual care (n = 408) in men and women receiving outpatient psychiatric care.

#### Behavioral outcomes

No studies provided data on contraception use or uptake of screening or treatment services. Seven studies assessed the total number of sex partners in the past 30 days [32], six weeks [33,36] or three months [28,34,35,37]. Two studies [28,32] found a significant reduction in the number of sex partners (n = 512), whereas the remaining studies [33-37] found no significant effect compared with usual care (n = 894).

Six studies reported data on unprotected intercourse. One study [32] observed significant reductions in rates of unprotected sex (anal, vaginal or oral) compared with usual care (n = 104) in the past month. Five studies assessed sexual risk behavior using the vaginal episode equivalent (VEE) score (a weighted measure of transmission risk from unprotected oral, anal, or vaginal intercourse) [39]. Whilst two studies [33,36] found a significant improvement in the VEE scores (n = 316) over the previous six weeks, three studies observed no significant improvements either in the past six weeks [25] or three months [26,29] compared with standard care (n = 320). Five studies assessed condom use (in the past 30 days [30], six weeks [33,36] or three months) [34,35,37] and one study evaluated condom use skills [34]. Two studies [30,36] observed a significant increase in the number of times condoms were used in sexual intercourse (n = 111). Similarly, two studies [33,35] found that more participants engaged (i.e. proportion of all encounters) in condom-protected intercourse (anal [33] or vaginal) [33,35] compared with usual care (n = 446); however, the effect was more pronounced in sex with women than men. One study [34] found a significant improvement in condom use skills (ability to properly enact steps in correctly placing a condom on a penis model), particularly in males, compared with usual care (n = 290). Although the majority of participants were of African-American origin in seven studies (range from 51% [35] to 72.4%) [37] only one study provided subgroup analysis data by ethnicity. This study [37] found that African-Americans in the intervention group reported significantly greater condom use (self-reported) than those in the control group; however, the intervention effect was not significant for Hispanic, Caucasian or other participants (n = 99).

#### Proxy outcomes

No studies evaluated barriers to, and facilitators of, sexual health promotion interventions for people with SMI. Seven studies provided data on knowledge, attitudes and beliefs, intentions and skills, which are important mediators of effect. Although no improvement was found in one study [34] (n = 290), six studies [28,30-32,35,38] (n = 800) observed an increase in HIV risk knowledge (in one of these studies this benefit was significant in men only, n = 189) [35]. Two studies [28,35] found significant improvements in attitudes for condom use (n = 597) when a HIV risk reduction programme was compared to usual care (in one of these studies this benefit was significant in women only but was not sustained beyond 12 months, n = 189) [35]. Behavioral intentions such as more frequent condom use or change in sexual risk behavior were improved in three studies [28,30,34] (n = 750). In contrast, no significant improvements were observed in two studies [35,38] (n = 209). Compared with usual care, two studies [28,38] observed a significant improvement in behavioral skills following sexual assertiveness training (n = 20) [38] or role-play simulations (n = 408) [28]. A detailed summary of the results for each included study is available in Kaltenthaler et al [40].

## Discussion

People with SMI are a high-risk population for rates of blood borne viruses, including HIV and Hepatitis C compared with the general population. This systematic review examined the evidence on the effectiveness of sexual health risk reduction interventions for people with SMI compared with usual care and their applicability to the UK setting. We found no clear and consistent evidence across all studies for reductions in the total number of sex partners or improved behavioral intentions in sexual risk behavior. Although data were limited, positive effects were generally observed in condom use, condom protected intercourse and on measures of HIV knowledge, attitudes to condom use and sexual behaviors and practices. These results should be viewed cautiously as most positive findings were not consistently sustained in some long-term (12 months or more) follow-up studies [35,36] and the low to moderate methodological quality of the majority of studies suggests the potential for bias in the study results. Despite the mixed findings of this systematic review, the results are broadly consistent with those of other reviews, but go beyond them because it includes data from eight additional studies [25,26,28,29,31,33,34,37] and also includes a published search strategy (for reproducibility) and an assessment on the risk of bias, which were absent in existing reviews. Johnson-Masotti et al. [41] found limited success (positive effects were generally in studies with small sample sizes) of HIV prevention interventions at helping people with SMI reduce their HIV risk behavior. Whereas, Higgins et al. [8] found that people experiencing SMI who attended sexual health education programmes (which focused on topics such as HIV and other sexually transmitted diseases, negotiating safe sex and skill development in condom use and were facilitated in a sensitive and supportive manner) benefited and produced a reduction in sexual risk behavior as opposed to complete cessation. In addition, small-group interventions combining information giving, motivational exercises and skills acquisition were found to be effective in reducing sexual risk behavior and raise awareness of personal risk behavior. However, one-to-one teaching may also be preferred for some topics by some people.

The literature available was limited in quantity and heterogeneous with respect to study populations, content and duration of interventions and comparators and assessment of sexual health related outcomes. It is also worth drawing attention to other limitations. First, although an extensive literature search was conducted, it is possible that some relevant studies may have been missed. However, such omissions are likely to have been minimal, as the search included all identifiable publications in the grey literature (including contact with clinical experts in the field). Second, although studies from developing countries were excluded after the full text review stage, the core features of sexual health risk reduction interventions for people with SMI in this setting could be relevant and considered for adaptation in the UK; however, no relevant studies were found. Moreover, all identified and included studies were based in the USA, and included participants from diverse ethnic backgrounds (seven studies predominantly included participants of African-American origin) [25-27,33-37]. As a result, the transferability of this review to European settings such as the UK, which has a different multicultural society with different values and customs, is questionable. Third, most of the studies included in the systematic review included participants with major depression, which was not included in the definition of SMI used for this review (and that of the UK Department of Health strategy documents) [1,42]. Patients with major depression have different needs and risk and may respond differently to interventions than those with bipolar disorder or schizophrenia or indeed there may be differences between these latter diagnoses. However, it was not possible to differentiate results for patients with different diagnoses. In addition, the majority of studies recruited individuals from outpatient psychiatric centers where they were receiving treatment, thus the findings may not be generalizable to people with SMI who do not seek or receive specialist mental health care [43]. Fourth, sexual health risk reduction interventions were heterogeneous in terms of content, duration, how they were delivered and by whom; whereas usual care often included components of the intervention such as education. Consequently, uncertainties remain around the beneficial components of sexual health risk reduction programs. Fifth, most outcomes were based on the participants self-reported sexual risk behavior so this may be subject to possible response and recall bias. These and other limitations make it difficult to assess the true magnitude and direction of effect of the sexual health risk reduction interventions.

Although none of the studies reported that they had been designed to have adequate power to assess differences in subgroups, some reported differences in results between male and female participants. This issue needs to be explored further as there may be real differences between male and female responses to sexual health risk reduction interventions. In addition, there was a dearth of evidence on the relationship between education levels and outcomes, intervention acceptability from participants or those delivering the service, feasibility of the interventions and how much it would cost to implement. We would also note, in relation to HIV, that there are inherent limitations of the review methodology within a changing health domain may mean much of the work in this area is becoming dated [44]. Within the last year new biomedical approaches to HIV prevention, such as pre-exposure prophylaxis (PrEP) have become available in the USA, and in the UK it is proposed that home testing for HIV will be legalized in early 2014. New opportunities for intervention delivery, particularly technologically-based, exist, with feasibility, acceptability and effectiveness evaluated [45-47]. A recent systematic review of interactive computer-based interventions (delivered via the internet or other technology such as interactive television, mobile phone, CD-ROM and handheld computers) for sexual health promotion showed that computer-based interventions were effective tools for learning about sexual health and feasible in a variety of settings [48]. However, evidence that people with SMI are able to engage with technologically-based interventions (e.g. computer and mobile) to improve mental health services, is limited [49,50]. As a result, the current evidence base may now fail to adequately address the complexity and challenges of delivering sexual health improvement intervention modalities, particularly to patients with SMI. It remains important to note that new interventions have been developed from those conducted in the pre-highly active antiretroviral era (HAART), and are rated as ‘best evidence’ by the CDC, such as Kelly’s ‘Popular Opinion Leader’ intervention adapted for African-American men who have sex with men [51].

Whilst there have been many initiatives in the UK to make health and social care more responsive and inclusive, the sexual health needs of individuals with psychosis appear to remain marginalized and neglected [52]. Despite variations in practice across the UK, the majority of mental health workers who are involved in the care of people with mental health problems (including SMI) in the UK do not provide sexual health promotion activities (including education) even though it is within the domain of their profession [53,54]. In contrast, in the Shetland Isles, Scotland, all mental health staff working with people with psychiatric illnesses receive sexual health training [55] although it is not clear exactly what this training includes and whether or not this varies with different types of staff.

Whilst people with SMI are able to engage in complex health interventions and benefit from them, several factors are relevant to implementing sexual health risk reduction interventions for people with SMI in the UK. These include consideration of who will deliver the interventions, where they will be delivered, whether sexual health risk reduction interventions could be integrated into the provision of current care and, if so how practitioners will be educated to deliver the interventions. A current on-going study [56] may provide some evidence on how physical co-morbidity in people with SMI could be addressed by community mental health nurses in the UK. The location of sexual health services for people with SMI and delivery of sexual health risk reduction interventions to groups or individuals will have resource implications and the potential to impact on effectiveness of the intervention. The long term impact of interventions, as well as the impact of ‘timed booster education sessions’ also needs to be taken into account as some of the included studies in the review showed a diminished impact at follow-up. Higgins et al. [8] suggests that sexual health education needs to be an ongoing process with sustained input, rather than a single intervention.

## Conclusions

Despite the limitations to the review, this is the first comprehensive systematic review of sexual health risk reduction interventions for people with SMI. Previous reviews in this area have focused on HIV [2,57]. The large between study variability (especially in the populations, interventions, comparators, and reported outcomes) and mixed results, provide insufficient evidence at present to fully support or reject the identified sexual health risk reduction interventions for people with SMI. Given the lack of UK based studies, well designed trials of sexual health improvement interventions for people with SMI are warranted including training and support for staff implementing sexual health risk reduction interventions and an assessment of location and costs of proposed services. In addition, patient acceptability of proposed interventions also needs to be given careful consideration.

## Additional file

Additional file 1:
**Literature search strategy, a MEDLINE example.**
