# Supplementary material for: Sexual health risk reduction interventions for people with severe mental illness: a systematic review
Source: BMC Public Health. 2015 Feb 12;15:138. doi: 10.1186/s12889-015-1448-4 (PMC4330652; doi:10.1186/s12889-015-1448-4)
Supplement: Additional file 1: — Literature search strategy, a MEDLINE example. [file 12889_2015_1448_MOESM1_ESM.docx]

**Supplementary material: Literature search strategy, a MEDLINE example**

| Database searched: | Ovid MEDLINE(R) In-Process & Other Non-Indexed Citations and Ovid MEDLINE(R) |
| --- | --- |
| Platform or provider used: | Ovid SP |
| Date of coverage: | 1948 to August 2014 |
| Search undertaken: | August 2014 |
|  |  |

1. ((chronic$ or sever$ or persist$) and mental$ and (ill$ or disorder$)).mp.

2. exp Schizophrenia/

3. (schizo$ or hebephreni$ or oligophreni$ or psychotic$ or psychosis or psychoses).tw.

4. Paranoid Disorders/

5. exp Psychotic Disorders/

6. (paranoia or paranoid disorders or psychotic disorders or psychosis).tw.

7. exp Bipolar Disorder/

8. ((bipolar or bi polar) adj5 (disorder$ or depress$)).tw.

9. (hypomania$ or mania$ or manic$).tw.

10. (((cyclothymi$ or rapid or ultradian) adj5 cycl$) or RCBD).tw.

11. or/1-10

12. exp Sexual Behavior/

13. (sex* and (health or safe or safer or unsafe or risk or high-risk or unprotected or abstinence or behaviour* or behavior* or activit* or partner*)).mp.

14. exp Sexually Transmitted Diseases/

15. ((STI or STIs or STD or STDs) and (incidence or prevalen* or prevent* or control* or risk* or reduc*)).mp.

16. ((sexually transmitted disease* or sexually transmitted infection*) and (incidence or prevalen* or prevent* or control* or risk* or reduc*)).mp.

17. or/12-16

18. Randomized controlled trials as Topic/

19. Randomized controlled trial/

20. Random allocation/

21. randomized controlled trial.pt.

22. Double blind method/

23. Single blind method/

24. Clinical trial/

25. exp Clinical Trials as Topic/

26. controlled clinical trial.pt.

27. or/18-26

28. (clinic$ adj25 trial$).ti,ab.

29. ((singl$ or doubl$ or treb$ or tripl$) adj (blind$ or mask$)).tw.

30. Placebos/

31. Placebo$.tw.

32. (allocated adj2 random).tw.

33. or/28-32

34. 27 or 33

35. Case report.tw.

36. Letter/

37. Historical article/

38. 35 or 36 or 37

39. exp Animals/

40. Humans/

41. 39 not (39 and 40)

42. 38 or 41

43. 34 not 42

44. 11 and 17 and 43
